# Axo-Glia Interaction Preceding CNS Myelination Is Regulated by Bidirectional Eph-Ephrin Signaling

**DOI:** 10.1177/1759091415602859

**Published:** 2015-09-04

**Authors:** Cecilie Linneberg, Mette Harboe, Lisbeth S. Laursen

**Affiliations:** 1 Department of Molecular Biology and Genetics, Aarhus University, Gustav Wieds Vej, Aarhus, Denmark

**Keywords:** Myelin, oligodendrocyte, Eph, ephrin, axo-glia interaction, integrin

## Abstract

In the central nervous system, myelination of axons is required to ensure fast saltatory conduction and for survival of neurons. However, not all axons are myelinated, and the molecular mechanisms involved in guiding the oligodendrocyte processes toward the axons to be myelinated are not well understood. Only a few negative or positive guidance clues that are involved in regulating axo-glia interaction prior to myelination have been identified. One example is laminin, known to be required for early axo-glia interaction, which functions through α6β1 integrin. Here, we identify the Eph-ephrin family of guidance receptors as novel regulators of the initial axo-glia interaction, preceding myelination. We demonstrate that so-called forward and reverse signaling, mediated by members of both Eph and ephrin subfamilies, has distinct and opposing effects on processes extension and myelin sheet formation. EphA forward signaling inhibits oligodendrocyte process extension and myelin sheet formation, and blocking of bidirectional signaling through this receptor enhances myelination. Similarly, EphB forward signaling also reduces myelin membrane formation, but in contrast to EphA forward signaling, this occurs in an integrin-dependent manner, which can be reversed by overexpression of a constitutive active β1-integrin. Furthermore, ephrin-B reverse signaling induced by EphA4 or EphB1 enhances myelin sheet formation. Combined, this suggests that the Eph-ephrin receptors are important mediators of bidirectional signaling between axons and oligodendrocytes. It further implies that balancing Eph-ephrin forward and reverse signaling is important in the selection process of axons to be myelinated.

## Introduction

Myelination of axons in the central nervous system by oligodendrocytes is important, not only to ensure fast transmission of signals but also to provide metabolic support for the neurons (Funfschilling et al., 2012; Lee et al., 2012). Several lines of evidence indicate that myelination is a highly regulated process in which local axo-glia signaling is required. First, the thickness of the individual myelin sheath correlates closely with the axonal diameter, and each oligodendrocyte can myelinate multiple axons (Friede, 1972; Murtie et al., 2007). Second, only axons above a certain diameter are myelinated (Waxman and Bennett, 1972), and third, recent data suggest that internodes are not evenly spaced throughout the length of the axon (Tomassy et al., 2014).

Live imaging in zebrafish has shown that oligodendrocytes go through a dynamic period of process extensions and retractions prior to the final selection of the axons to be myelinated (Kirby et al., 2006). However, following the initial wrapping of the oligodendrocyte processes around the axon, very few retractions are observed (Czopka et al., 2013), suggesting the existence of a narrow time window in which the axons are selected. However, the precise mechanism by which adhesion molecules mediating bidirectional axo-glia communication guide this selection process is still unclear. A limited number of cell surface molecules has been suggested to participate in the initial interaction. For example, downregulation of PSA-NCAM on axons and oligodendrocyte precursor cells (OPCs) is required prior to initiation of myelination (Charles et al., 2000; Fewou et al., 2007). Similarly, downregulation of Lingo signaling in oligodendrocytes is also required to induce oligodendrocyte differentiation and initiation of myelination (Mi et al., 2005, 2007). However, until recently, when overexpression of NRGI Type III was shown to enhance myelination (Brinkmann et al., 2008), and signaling through β1-integrin was found to be involved in initiation of myelination (Camara et al., 2009), no positive guidance clues had been revealed. The latter finding implies that molecules regulating integrin activation may be involved in the determination of when the oligodendrocyte will form permanent contacts with a given axon.

During development, the Eph-ephrin system comprises important mediators of bidirectional signaling between neighboring cells. Depending on the cellular context, the multiple possible interactions between Eph and ephrin receptors result in cell–cell adhesion or repulsion (Arvanitis and Davy, 2008; Klein, 2012). The Eph receptors constitute the largest family of tyrosine kinase receptors known and are, based on structural similarities and ligand binding preferences, divided into two subfamilies, EphA (1–8) and EphB (1–4). EphAs bind preferentially to the glycophosphatidylinositol (GPI)-anchored ephrin-As, whereas EphBs bind to the transmembrane ephrin-Bs, EphA4 being an exception, which binds members of both subfamilies (Bowden et al., 2009; Gale et al., 1996). Activation of the Eph receptors by ephrins is referred to as forward signaling. At the same time, binding of Eph to ephrin induces reverse signaling, even though this reverse signaling mechanism is less clear. Hence, the Eph-ephrin system signals bidirectionally between adjacent cells (Lisabeth et al., 2013).

In the nervous system, the Eph-ephrin receptors are known for their role in axonal guidance (Feldheim et al., 2000; Hindges et al., 2002; Kania and Jessell, 2003; Luria et al., 2008), and more recent data has also shown that several family members are intimately involved in the regulation of neuronal progenitor positioning (Arvanitis et al., 2013; Dimidschstein et al., 2013) and in controlling synapse formation and plasticity (Grunwald et al., 2004; Kayser et al., 2008; Lim et al., 2008). In addition, in other cell types, both forward and reverse signaling have been suggested to regulate cell adhesion by controlling integrin activation (Arvanitis et al., 2013; Bourgin et al., 2007; Huynh-Do et al., 1999, 2002; Miao et al., 2000). Mouse OPCs express ephrin-B2, ephrin-B3 and ephrin-A5, and reverse signaling, induced by Eph receptors present on axons, has been speculated to guide migration of these cells (Prestoz et al., 2004). Similarly, ephrin-B1 has been suggested to inhibit migration of OPCs (Cohen, 2005). However, a role of the Eph-ephrin system in axo-glia interactions has not been reported.

We here aim to test the hypothesis that Eph-ephrin signaling is important for the initial axo-glia interaction that can lead to myelination. We further hypothesize that such an effect is mediated by controlling the activational status of β1-integrins.

## Material and Methods

### Cell Culture and Transfection

Primary OPCs were obtained as described (McCarthy and de Vellis, 1980; Milner and Ffrench-Constant, 1994). Briefly, dissociated rat neonatal cortices were cultured at 37℃ in 5.0% CO_2_ in Dulbecco’s modified Eagle’s medium (DMEM) with 10% fetal calf serum (FCS) and penicillin/streptomycin in Poly-D-lysine (PDL)-coated flasks. By Day 10, cultures consist of OPCs and microglia growing on an astrocyte monolayer (so-called *mixed glia cultures*). Cell populations enriched for OPCs were acquired by mechanically shaking them off the surface of the astrocytes and then removing microglia by differential adhesion.

For transfection, OPCs were cultivated in Sato medium (Bottenstein and Sato, 1979) with 0.5% FCS, including Platelet-derived growth factor (PDGF) (10 ng/mL) and Fibroblast growth factor (FGF) (10 ng/mL). Transfection of the OPCs with plasmids was performed with FuGENE 6 (Promega). The day after transfection, cells were trypsinated and replated on PDL- or substrate-coated coverslips in 12 well plates. Cells were allowed to differentiate in Sato medium supplemented with 0.5% FCS, and penicillin/streptomycin and after 2–5 days they were fixed in 4% paraformaldehyde (PFA).

For differentiation of OPCs on different substrates, coverslips were first coated with PDL, followed by coating with recombinant fc-fused ephrins or Eph-receptors, obtained from R&D systems, at a concentration of 2 µg/mL, or with a combination of Laminin 1 and 2 (Sigma Aldrich) 10 µg/mL. OPCs were plated at a density of 75,000 cells per 18 mm coverslips in 12 well plates.

For analysis of Eph-A2 phosphorylation, OPCs were plated onto PDL-coated six well plates and allowed to differentiate for 24 hr prior to stimulation with soluble ephrin-A1 (1 µg/mL) or ephrin-A1, preclustered with Fc-Ab (1 µg/mL) for 30 min prior to lysis as described below.

Myelinating cocultures of oligodendrocytes and Dorsal root ganglion (DRG) neurons were generated as described earlier (Adamo et al., 2001; Laursen et al., 2009; Wang et al., 2007). In brief, embryonic DRG neurons were isolated from E15-E16 rats and dissociated with papain (1.2 U/mL; Sigma), L-cysteine (0.24 mg/mL; Sigma), and DNase I (0.40 mg/mL; Sigma) for 60 min at 37℃. The dissociated cells were plated at a density of 200 × 10^3^ cells per coverslip (18 mm) coated with PDL and growth factor-reduced matrigel (BD Biosciences). The neurons were then cultured for 17 days in DMEM (Sigma), 10% FCS (Gibco, Invitrogen) in the presence of nerve growth factor (NGF; 100 ng/mL; Serotec). To remove contaminating cells, the cultures were pulsed three times for 2 days each with fluorodeoxyuridine (10 IU; Sigma) at Days 2, 5, and 8 after seeding. After 17 days, the medium was changed to a 50:50 mixture of Sato’s modification of DMEM (Sato; Bottenstein and Sato, 1979; Milner and Ffrench-Constant, 1994) and Neurobasal (Gibco), supplemented with 2% B27 (Gibco, Invitrogen), N-acetyl cysteine (5 µg/mL; Sigma), and D-biotin (10 ng/mL), and 100 × 10^3^ OPCs were added to each well. After 18 days in coculture, the cells were fixed in 4% PFA.

### Western Blotting and Protein Analysis

Cells were washed in 1 × Phosphate buffered saline (PBS) and lysed in ice-cold Radio-immunoprecipitation Assay (RIPA) buffer (Sigma Aldrich) supplemented with proteinase and phosphatase inhibitors for 15 min on ice. The cells were scraped off and transferred to microtubes. Lysates were cleared by centrifugation for 15 min at 4℃. Proteins were separated by SDS-PAGE and blotted onto polyvinylidene difluoride (PVDF) membrane (Millipore). Membranes were dried and blocked in 2% Tween-20, followed by overnight incubation with primary antibodies in 20 mM Tris, 150 mM NaCl, containing 0.1% Tween-20, pH 7.6 (TBS-T). Membranes were washed in TBS-T and incubated for 1 hr with Horseradish peroxidase (HRP)-conjugated secondary antibodies (GE Healthcare), washed again in TBS-T, and developed using Enhance chemiluminescense (ECL)-Prime (GE Healthcare). Images were acquired with an Image Quant Las 4000.

### Immunoprecipitation

Cell lysates were generated as described above following stimulation with PBS (control), soluble ephrin-A1, ephrin-A1 preclustered with Fc-Ab, soluble ephrin-B2, or ephrin-B2 preclustered with Fc-Ab. Precleared lysates were incubated with PY99 beads (Santa Cruz, Biotechnology) or protein G-coupled EphA4 antibodies (Abcam) over night at 4℃, followed by washing three times in RIPA buffer supplemented with phosphatase and proteinase inhibitor, and finally resuspened in sample buffer and subjected to Western blotting.

### Immunocytochemistry and Microscopy

Cells were fixed with 4% PFA, blocked with PBS containing 10% goat serum, 0.1% Triton-X 100 for 20 min, and incubated with primary antibodies diluted in blocking buffer for 2 hr. After washing with PBS, cells were incubated with secondary antibodies diluted in blocking buffer for 1 hr, washed with PBS with Hoechst nuclear stain (Invitrogen) included in the last washing buffer, and finally the coverslips were mounted on slides with Fluoromount-G (SouthernBiotech). Antibodies against EphB1, EphA2, EphA4, and ephrin-B1 and phospho-tyrosine were from Santa Cruz biotechnology, the antibody against ephrin-B2 was from Novus Biologicals, the antibodies against ephrin-B3 and Phospho-EphB were from Abcam, the antibodies against phospho-EphA2 and phospho-FAK were from Cell Signaling Technology, the antibody against Enhanced green fluorescent protein (EGFP) was from Life technologies, the antibody against neurofilament was from EnCor Biotechnology, the antibody against Myelin basic protein (MBP) was from Serotec, and the antibodies against Neural/glial antigen 2 (NG2) and Myelin associated glycoprotein (MAG) were from Millipore. All secondary antibodies were from Invitrogen. Images were acquired on a Zeiss Axio Observer.Z1 Apotome 2 or a Zeiss Axio Observer D1 microscope. Image analysis was performed with Image-J and Adobe Photoshop.

### Plasmid DNA

EGFP-C1 (Clontech) was used in combination with an empty vector, pcDNA3.1 (Invitrogen), wild-type β1-integrin inserted into pcDNA3.1, or a constitutive active integrin variant, in which the aspartic acid involved in formation of a salt bridge to a arginine in the alpha-subunit was exchange with a arginine, describe in (Hughes et al., 1996; Laursen et al., 2011), also inserted in to pcDNA 3.1.

### RNA Isolation and QRT-PCR

RNA was isolated from oligodendrocytes generated from OPCs differentiated for 1 to 4 days *in vitro* using a RNA easy kit from Qiagen. The RNA was reverse transcribed and amplified with the Brilliant III Ultra-Fast SYBR Green QRT-PCR (Stratagene), and analyzed on a Stratagene Mx3005P. The following Q-PCR primers were used: MBP: 5′-ACTTGGCCACGCAAACTACC-3′ and 5′-GGGTGTACGAGGTGTCACAA-3′, actin: 5′-AGCCATGTACGTAGCCATCC-3′ and 5′-CTCTCAGCTGTGGTGGTGAA-3′, EphA2: 5′-CCTGCAAAGGACCCAGCTAA-3′ and 5′-CACAGCCAAGCATCCTGAGA-3′, EphA4: 5′-TATACTACCAGGGGCGGCAA-3′ and 5′-AACTGATGGAGGGCAATGGG-3′, EphB1: 5′-TCAGTGGCAAGATGTGCTTC-3′ and 5′-GCCTGTGCTGTAATGCTGAA-3′, EphB2: 5′-CCAGCGCTCTGGGTGGGAAG-3′ and 5′-GGGCGGAGGTAGCCGGTAGT-3′, ephrin-A1: 5′-CCCACATTACGAGGACGACT-3′ and 5′-GTGAAGCGCTGGAATTTCTC-3′, ephrin-A5: 5′-GCCTCACTCTCCAAACGGAC-3′ and 5′-GTACGGTGTCATCTGCTGGTT-3′, ephrin-B1: 5′-GGCAAGCATGAGACTGTGAAC-3′ and 5′-TAGGGTACTGAGCGAGAGGG-3′, ephrin-B2: 5′-TCCCTTTGTGAAGCCAAATC-3′ and 5′-GTCTCCTGCGGTACTTGAGC-3′, and ephrin-B3: 5′-GACAGCATACCAGGTGACCC-3′ and 5′-CAGAGACCCTCCTCTCCCAA-3′. The fold increase in mRNA relative to cells differentiated for 1 day was calculated as described (Schmittgen and Livak, 2008) using actin as the internal control.

## Results

### Expression of Both Ephrin and Eph Receptors Is Regulated during Oligodendrocyte Differentiation

**Figure 1.**
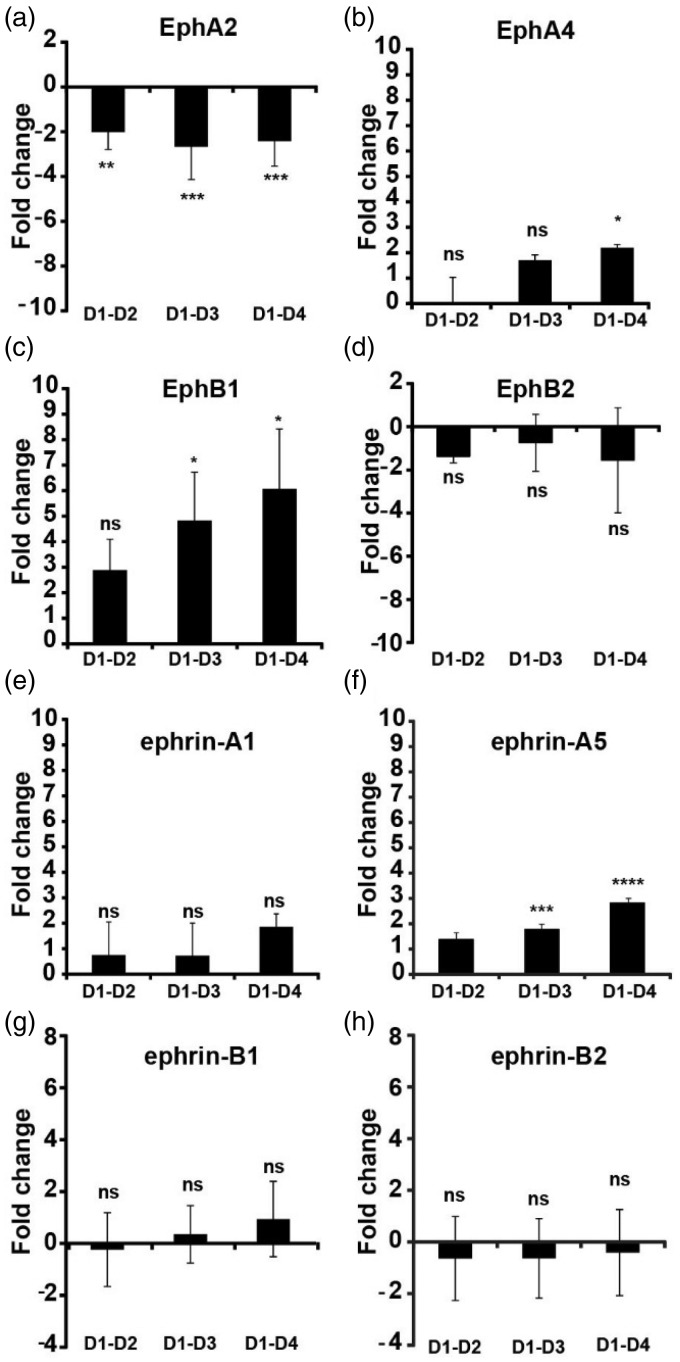
Expression of Ephs and ephrins in oligodendrocytes differentiated for 1 to 4 days. mRNA was isolated from oligodendrocytes generated from oligodendrocyte precursors allowed to differentiate for 1 to 4 days *in vitro.* The mRNA levels were measured by QRT-PCR, and the changes in mRNA expression during differentiation were expressed as fold changes relative to the mRNA level at Day 1 in culture, using actin as an internal control. (a) EphA2. (b) EphA4. (c) EphB1. (d) EphB2. (e) Ephrin-A1. (f) Ephrin-A5. (g) Ephrin-B1. (h) Ephrin-B2. Values are displayed as mean ± *SD* of samples from at least three independently generated mRNA preparations, each run in triplicate. Statistical differences were tested by one-way analysis of variance followed by a Dunnett’s posttest. (*p < .05; **p < .01; ***p < .001; ****p < .0001.)

Initially, we tested whether members of both the A and B subfamilies of ephrin and Eph receptors are expressed in OPCs, and whether their expression level changed during differentiation. We selected widely studied members of the four families to which suitable antibodies were available. The mRNA levels of EphA2, -A4, -B1, and -B2, and ephrin-A1, -A5, -B1, -B2, and -B3 were assessed in OPCs and in oligodendrocytes at different stages of differentiation relative to that of actin mRNA, which remains unchanged during the 4 days of differentiation *in vitro*. All of the tested Eph and ephrin transcripts were found to be expressed in both OPCs (Day 1) and more mature cells (Day 2–Day 4), but the mRNA level of only a few of them changed during differentiation. Detection of ephrin-B3 mRNA was, however, only possible with increased amount of input RNA (Supplementary Figure 1(a)). Of the Ephs, minor changes were detected for EphA2 (Figure 1(a)) and EphA4 (Figure 1(b)), which were down and upregulated approx. twofold, respectively. EphB1 displayed a marked upregulation of approx. sixfold (Figure 1(c)). In contrast, only one of the tested ephrins, ephrin-A5, showed a significant change in expression, an approx. threefold upregulation (Figure 1(e)).

**Figure 2.**
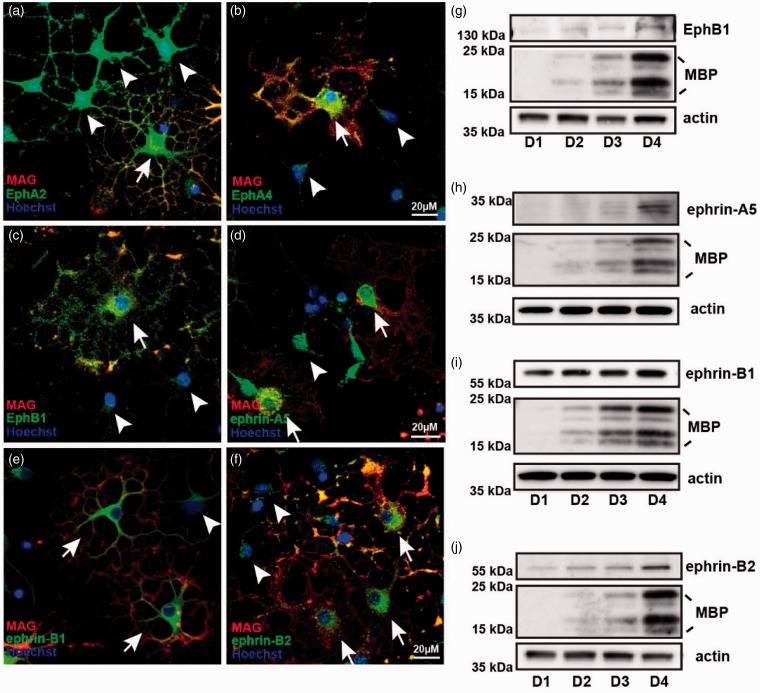
EphB1, EphA4, ephrin-B1, and ephrin-B2 are upregulated in mature oligodendrocytes. Immunocytochemistry of oligodendrocytes generated from oligodendrocyte precursors allowed to differentiate for 3 days *in vitro*. (a) Cells stained for EphA2 (green), MAG (red), and Hoechst (blue). (b) Cells stained for EphA4 (green), MAG (red), and Hoechst (blue). (c) Cells stained for EphB1 (green), MAG (red), and Hoechst (blue). (d) Cells stained for ephrin-A5 (green), MAG (red), and Hoechst (blue). (e) Cells stained for ephrin-B1 (green), MAG (red), and Hoechst (blue). (f) Cells stained for ephrin-B2 (green), MAG (red), and Hoechst (blue). Immature (arrowheads) and mature (arrows) oligodendrocytes are pointed out. (g–j) Western blotting of lysates from oligodendrocytes generated from oligodendrocyte precursors allowed to differentiate for 1 to 4 days *in vitro,* (g) EphB1, (h) ephrin-A5, (i) ephrin-B1, (j) ephrin-B2. MBP and actin were used as differentiation and loading control respectively.

To further analyze the consequences of these observed changes in transcription, we also looked for changes at the protein level by immunocytochemistry and Western blotting. EphA2 was found to be expressed in both OPCs and mature MAG-positive oligodendrocytes (Figure 2(a)), but a clear downregulation could not be detected by immunocytochemistry. Similar to what was observed at the mRNA level, increased amounts of EphA4 and EphB1 receptors were detected in mature oligodendrocytes compared with OPCs (Figure 2(b), (c), and (g)). Also, ephrin-A5 was confirmed to be upregulated during oligodendrocyte differentiation (Figure 2(d) and (h)). Interestingly, a slight upregulation of ephrin-B1 (Figure 2(e) and (i)) and a more pronounced upregulation of ephrin-B2 (Figure 2(f) and (j)) were also observed in mature oligodendrocytes (D4), compared with immature cells (D1). The upregulation of ephrin-B1 and -B2 during differentiation is in contrast to what was observed at the mRNA level, suggesting that these transcripts may undergo posttranscriptional regulation. To summarize, the observed changes in expression of both Eph receptors and ephrins, as the OPCs differentiate into oligodendrocytes, indicate that this signaling system may play a role in coordinating oligodendrocyte differentiation and axo-glia interactions. Importantly, our finding that both receptors (Ephs) and ligands (ephrins) are present on the maturing oligodendrocytes show that both forward and reverse signaling is possible between oligodendrocyte processes and axons.

### Ephrin-A1 Reduces Process Outgrowth and Inhibits Myelin Sheet Formation

**Figure 3.**
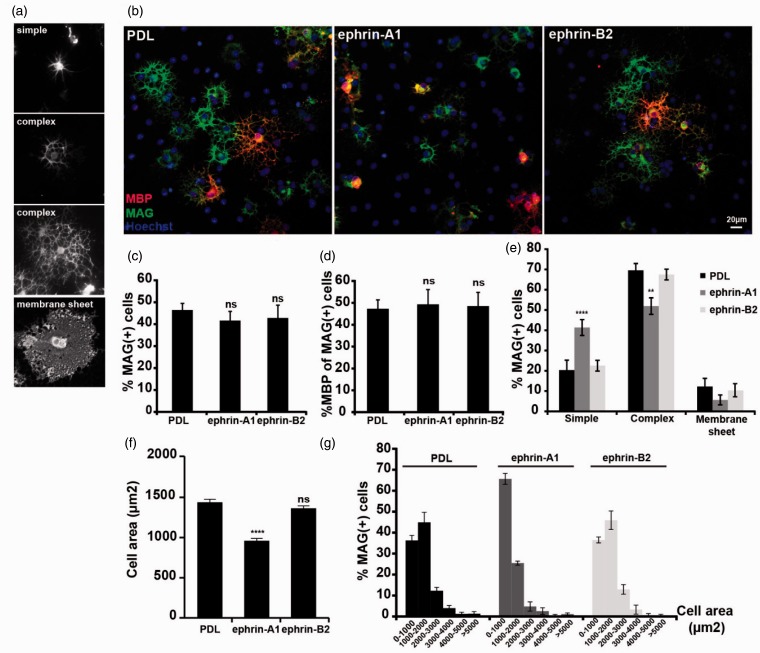
Ephrin-A1 inhibits process extension and the morphological differentiation of oligodendrocytes. Oligodendrocyte precursors were allowed to differentiate for 3 days *in vitro* on PDL, ephrin-A1, or ephrin-B2 substrates as indicated. (a) Examples of the different cell morphologies observed during differentiation of oligodendrocytes. Top panel shows cell with simple straight processes. Center panels show cells forming complex networks of branching processes. Bottom panel shows membrane sheet-forming cell. (b) Differentiation was analyzed after immunecytochemistry for MAG (green), MBP (red), and Hoechst (blue). Quantification of the experiment is present in the following three panels with means ± *SD* shown from at least 10 pictures from each of two coverslips (approx. 500 cells counted/coverslip) in three independent experiments. (c) Percentage of MAG + cells. (d) Percentage MBP + of MAG + cells. (e) The morphology of the MAG + cells was scored as “simple,” “complex,” and “membrane.” Statistical significance was analyzed by one-way analysis of variance (ANOVA) followed by Dunnett’s Multiple Comparison test (B and C) or two-way ANOVA followed by a Dunnett’s posttest (d). (*p < .05; **p < .01; ***p < .001.) The average cell sizes (f) and size distribution (g) of the cells cultured on the indicated substrates are shown. At least 150 cells from three independent experiments were analyzed, and average cell size ± *SEM* is displayed. Statistical significance was tested by one-way ANOVA followed by Tukey’s Multiple Comparison test (*ns* = nonsignificant; ****p < .0001). PDL: poly-D-lysine.

To delineate the role of Eph-ephrin signaling during the final stage of oligodendrocytes differentiation when myelin sheath formation is initiated, forward and reverse signaling must be analyzed separately. To do this, we first used a model system of primary rat OPCs, which, when differentiated *in vitro*, still undergo dramatic morphological changes resembling myelin sheath formation. During differentiation, the newly formed oligodendrocytes gradually change their morphology from cells with simple processes to cells with highly branched processes before they finally extend large membrane sheets (Figure 3(a)). In this process, the cells also start to express myelin-specific genes, such as MAG and MBP. We first analyzed the effect of EphA and EphB forward signaling by culturing OPCs on PDL, ephrin-A1, or ephrin-B2 substrates for 3 days. Hereafter, the expression of MAG and MBP, and the cell morphology were analyzed (Figure 3(b)–(d)). No changes in expression of neither MAG nor MBP were observed upon culture on ephrin-A1 or ephrin-B2 (Figure 3(c) and (d)). However, EphA forward signaling induced by ephrin-A1 resulted in a reduced ability of the oligodendrocytes to form complex branching networks and myelin sheets (Figure 3(e)), a similar effect was also observed with a higher concentration of ephrin-A1 and with ephrin-A5 (Supplemental Figure 2). In addition to the reduced branching complexity of the oligodendrocyte processes, the overall cell size was reduced (Figure 3(f) and (g)), suggesting that the ability of oligodendrocytes to extend processes is compromised upon culture on ephrin-A1. By contrast, no effect was observed for cells cultured on an ephrin-B2 substrate.

### EphB Forward Signaling Regulates the Activation Status of Β1 Integrin

**Figure 4.**
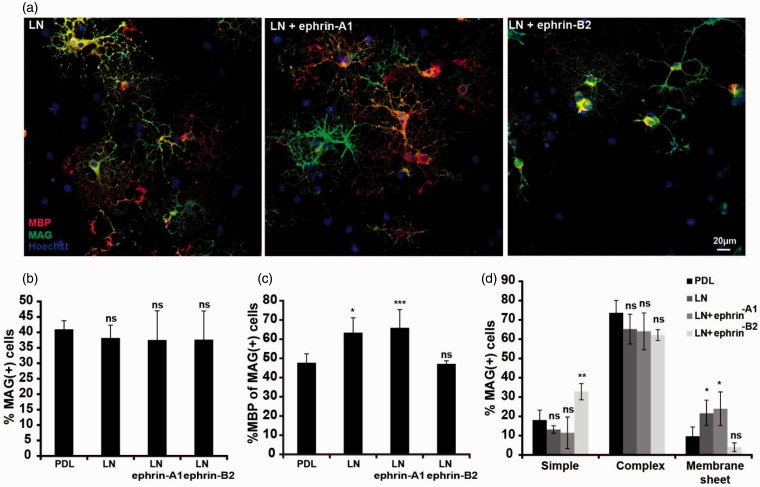
Ephrin-B2 inhibits a laminin-induced increase in MBP expression and myelin sheet formation. Oligodendrocyte precursors were allowed to differentiate for 3 days *in vitro* on a laminin or PDL substrate in the absence or presence of soluble ephrin-A1 or ephrin-B2, as indicated. (a) Differentiation was analyzed after immunocytochemistry for MAG (green), MBP (red), and Hoechst (blue). Quantification of the experiment is shown in the next three panels, with means ± *SD* shown from at least 10 pictures from each of two coverslips (approx. 500 cells counted/coverslip) in three independent experiments. (b) Percentage of MAG + cells. (c) Percentage MBP + of MAG + cells. (d) The morphology of the MAG + cells was scored as “simple,” “complex,” and “membrane.” Statistical significance was analyzed by one-way analysis of variance (ANOVA) followed by a Dunnett’s posttest (b and c) or two-way ANOVA followed by a Dunnett’s posttest (d). (*p < .05; **p < .01; ***p < .001; ****p < .0001.) PDL: poly-D-lysine.

In other cell systems, both EphA and EphB forward signaling have been shown to regulate integrin activation and cell adhesion (Bourgin et al., 2007; Huynh-Do et al., 1999; Miao et al., 2000). The α6β1 integrin ligand laminin is known to enhance the morphological differentiation of the oligodendrocytes and also to control the timing of MBP expression (Buttery and ffrench-Constant, 1999; Laursen et al., 2011). To analyze whether Eph signaling is required to regulate integrin activation in oligodendrocytes, the effect of soluble Ephrin-Fc variants on oligodendrocytes cultured on a laminin substrate was analyzed. Under these conditions, no effect of soluble ephrin-A1 was observed (Figure 4(a)–(d)), which might implicate that clustering of the ligand on the cell surface of neurons or at the bottom of a cell culture dish is required for efficient stimulation of the EphA receptors. By contrast, stimulation of OPCs with soluble ephrin-B2 reduced the expression of MBP to a level similar to cells cultured in the absence of laminin, without reducing the number of MAG-positive cells (Figure 4(b) and (c)). The number of oligodendrocytes, which forms myelin sheets in culture, was also decreased by ephrin-B2 (Figure 4(d)). This indicates that ephrin-B2 is involved in regulating the activation of β1-integrins.

**Figure 5.**
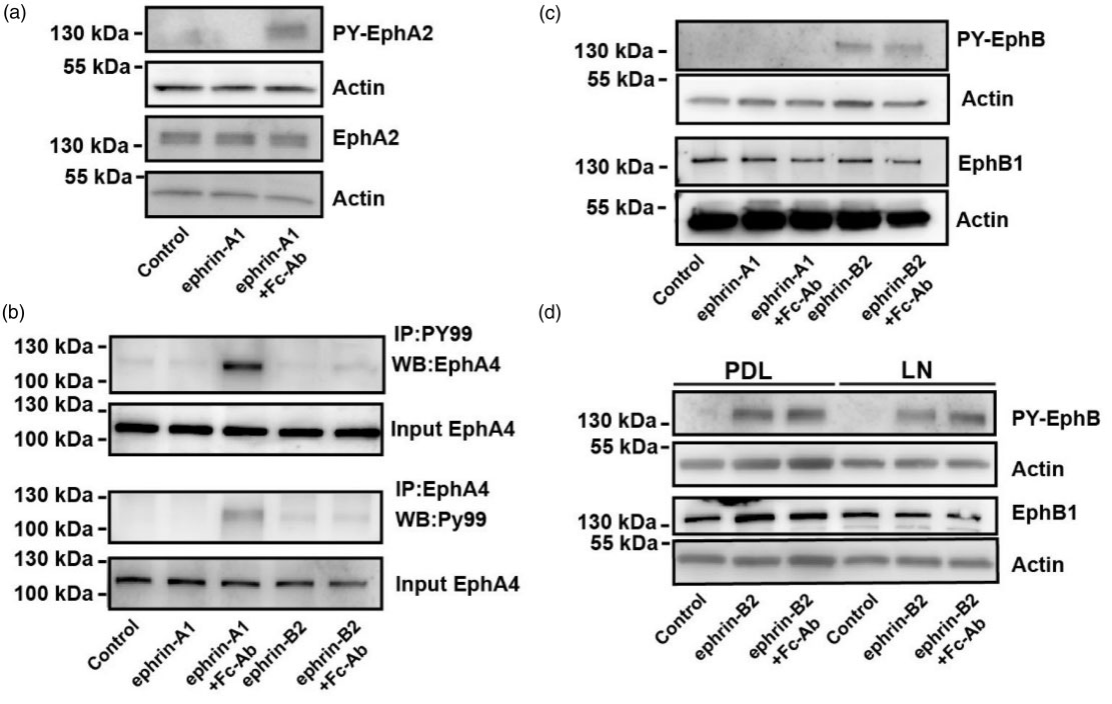
Clustering of ephrin-A1 is required for activation of EphA2 and -A4 in oligodendrocytes. Lysate from OPCs was generated following stimulation with PBS (control), soluble ephrin-A1, ephrin-A1 preclustered with Fc-Ab, soluble ephrin-B2, or ephrin-B2 preclustered with Fc-Ab, as indicated. (a) Lysates were analyzed by Western blotting using EphA2 phospho-tyrosine-specific antibodies or total EphA2 antibodies. Actin was used as the loading control. (b) Lysates were subjected to immunoprecipitation with phospho-tyrosine-specific antibodies and analyzed by EphA4 Western blotting (top panels), or immunoprecipitated with EphA4 antibodies and analyzed by PY99 Western blotting (bottom panels). Input of EphA4 was used as a control. (c) Lysates were analyzed by Western blotting using EphB1 phospho-tyrosine-specific antibodies or total EphB1 antibodies. Actin was used as the loading control. (d) Lysates from OPC cultured on PDL or laminin substrates were analyzed by Western blotting using EphB1 phospho-tyrosine-specific antibodies or total EphB1 antibodies. Actin was used as the loading control.

The ability of soluble ephrins to induce receptor activation is cell-type-specific and has been suggested to depend on the level of receptor expression (Davis et al., 1994; Janes et al., 2012). To confirm that the functional effects we observe with clustered ephrin-A1 and soluble ephrin-B2 correlate with Eph-receptor phosphorylation, we analyzed the phosphorylation status of the Eph-receptors following stimulation with soluble or preclustered ephrin-A1 or -B2. Indeed, EphA2 and EphA4 phosphorylation was only observed with preclustered ephrin-A1 (Figure 5(a) and (b)). In contrast, soluble ephrin-B2 was sufficient to induce EphB receptor phosphorylation (Figure 5(c)), and importantly, the concentration of ephrin-B2 used in these experiments was not sufficient to induce phosphorylation of EphA4 at the same level as clustered ephrin-A1 (Figure 5(b)). The latter probably reflects the approx. tenfold lower affinity of EphA4 for ephrin-B2 relative to ephrin-A1-EphA4 (Qin et al., 2010) or ephrin-B2-EphB2 (Ran et al., 2008). Furthermore, the ability of ephrin-B2 to induce EphB receptor phosphorylation was unchanged in cells cultured on laminin (Figure 5(d)), implicating that integrins are not regulating EphB signaling.

**Figure 6.**
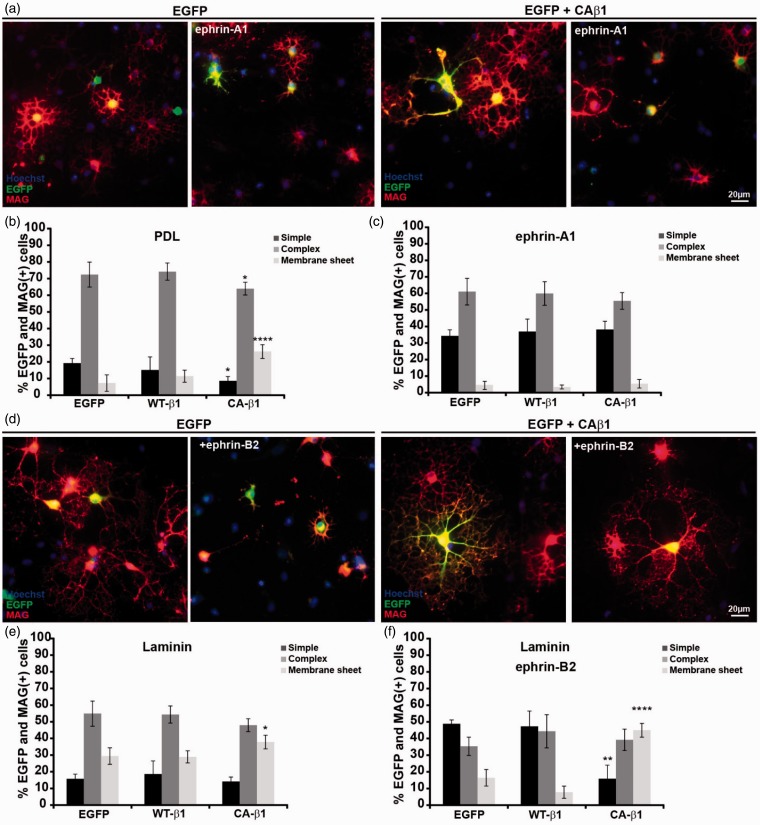
Ephrin-B2-induced inhibition of myelin sheet formation is integrin dependent, whereas ephrin-A1-induced inhibition is not. Oligodendrocytes generated from OPCs cotransfected with EGFP cDNA and empty vector, wild-type (WT), or constitutive active (CA) β1-integrin, as indicated. The cells were cultured on PDL, ephrin-A1, or laminin in the absence or presence of soluble ephrin-B2, as indicated. The cells were stained for EGFP (green) and MAG (red), and the morphology was scored as “simple,” “complex,” or “membrane” based on their staining for EGFP and MAG. At least 100 EGFP + cells were scored in each experiment. Results are an average of three independent experiments ± *SD* with statistical significance analyzed by two-way analysis of variance followed by a Dunnett’s posttest *p < .05; **p < .01; ***p < .001. (a) Cells cultured on PDL or ephrin-A as indicated. (b) Quantification of experiments for cells cultured on PDL. (c) Quantification of experiments for cells cultured on ephrin-A1. (d) Cells cultured on laminin in the absence or presence of ephrin-B2 as indicated. (e) Quantification of experiments for cells cultured on laminin. (f) Quantification of experiments for cells cultured on laminin in the presence of soluble ephrin-B2. PDL: poly-D-lysine.

To further test the ability of ephrin-A1 and -B2 to modulate integrin-mediated signaling, we repeated the experiment in oligodendrocytes transfected with wild-type β1 integrin (WT-β1) or a constitutive active β1-integrin (CA-β1). Overexpression of CA-β1 leads to enhanced Fak-phosphorylation in the transfected oligodendrocytes (Supplemental Figure 3), and an increase in the percentage of cells that forms myelin sheets in culture (Figure 6(a) and (b)), as reported previously (Laursen et al., 2011). Interestingly, the expression of the constitutive active integrin could not overcome the inhibitory effect of the ephrin-A1 substrate on oligodendrocyte process extension and myelin sheet formation (Figure 6(a) and (c)), suggesting that ephrin-A1 regulates these processes in a manner which does not depend on integrins. By contrast, when a similar experiment was performed with the addition of soluble ephrin-B2 on a laminin substrate, the cells expressing CA-β1 were not affected by ephrin-B2. However, the cells transfected with mock DNA or WT-β1 were still compromised in their ability to form myelin sheets (Figure 6(d)–(f)). This experiment substantiates that ephrin-B2 modulates activation of β1-integrins in the oligodendrocyte.

### Ephrin-B Reverse Signaling Enhances Myelin Sheet Formation in Oligodendrocytes

**Figure 7.**
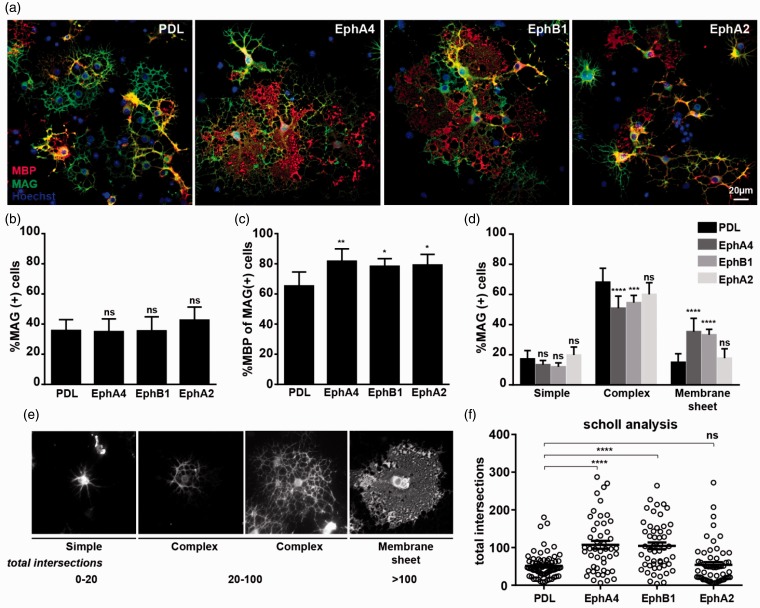
Ephrin-B reverse signaling enhances myelin sheet formation. Oligodendrocyte precursors were allowed to differentiate for 3 days *in vitro* on PDL, EphA4, EphB1, or EphA2, as indicated. (a) Differentiation was analyzed after immunecytochemistry for MAG (green), MBP (red), and Hoechst (blue). Quantification of the experiment is shown in the following three panels, with means ± *SD* shown from at least 10 pictures from each of two coverslips (approx. 400 cells counted/coverslip) in three independent experiments. (b) Percentage MAG + cells. (c) Percentage MBP + of MAG + cells. (d) The morphology of the MAG + cells was scored as “simple,” “complex,” and “membrane.” (e) Scholl analysis was used to measure the morphological differentiation of oligodendrocytes, an increase in the total number of intersections correlates with increased morphological complexity. (f) Scholl analysis of OPCs differentiated on substrates of PDL, EphA4, EphB1, and EphA2, as indicated. Statistical significance was analyzed by one-way (ANOVA) analysis of variance followed by a Tukey’s posttest (b, c, and f) or two-way ANOVA followed by a Tukey’s posttest (d). *p < .05; **p < .01; ***p < .001; ****p < .0001. PDL: poly-D-lysine.

EphA4 has previously been suggested to be implicated in myelination based on the expression pattern observed in hippocampal neurons at the onset of myelination (Tremblay et al., 2009). At this time point, the EphA4 receptors occur in large clusters where the oligodendrocyte processes are in close contact with the axon to be myelinated (Tremblay et al., 2009). To test whether EphA4-induced reverse signaling has an effect on oligodendrocyte differentiation and cell morphology, OPCs were cultured on an EphA4 substrate and allowed to differentiate for 3 days. Under these conditions, the number of MAG-positive cells was not significantly altered (Figure 7(a) and (b), but the percentage of MAG-positive cells expressing MBP was slightly increased (Figure 7(c)). Most interestingly, the presence of EphA4 leads to a profound increase in the number of oligodendrocytes forming very large membrane sheets (Figure 7(a) and (d)).

The EphA4 receptors are able to interact both with class A and B ephrins (Bowden et al., 2009; Gale et al., 1996). To further test whether the observed effect was a result of ephrin-A or ephrin-B reverse signaling, a similar experiment was carried out with cells cultured on substrates of either EphB1 or EphA2. Culturing the cells on EphB1 also increased the percentage of cells forming large myelin sheets, whereas cells cultured on EphA2 behaved similar to the control cells. The enhanced ability of cells cultured on EphA4 and EphB1 to form myelin sheets was further assessed by Scholl analysis. The measurement of total number of intersections in the Scholl analysis has previously been shown to correlate with increased cell size, branching complexity, and myelin sheet formation of the oligodendrocytes (Gensel et al., 2010). Our data show a similar correlation (Figure 7(e)). This analysis further confirmed the ability of EphA4 and EphB1 to stimulate myelin sheet formation in the oligodendrocytes (Figure 7(f)). Combined, our results suggest that the increased myelin sheet formation was a result of reverse signaling through ephrin-B.

### Interfering With Bidirectional Signaling Between EphA-Ephrin-A and EphB-Ephrin-B Has Opposing Effects on Myelination

To finally assess the importance of Eph-ephrin-mediated bidirectional signaling between neurons and oligodendrocytes, we used soluble ephrin-A1, ephrin-B2, and EphB1 variants in a coculture system of oligodendrocytes and DRG neurons. The DRG neurons express several Ephs and ephrins from both subfamilies, supporting that bidirectional signaling would indeed be possible in these cultures (Table 1).

**Table 1.** Ephrins and Ephs Expressed in DRG Neurons.

Ephs	EphA2, EphA4, EphB1, and EphB2
Ephrins	ephrin-A1, ephrin-A5, ephrin-B1, ephrin-B2, and ephrin-B3

A subset of Ephs and ephrins was tested by QRT-PCR for their expression in DRG neurons cultured for 14 days *in vitro*.

**Figure 8.**
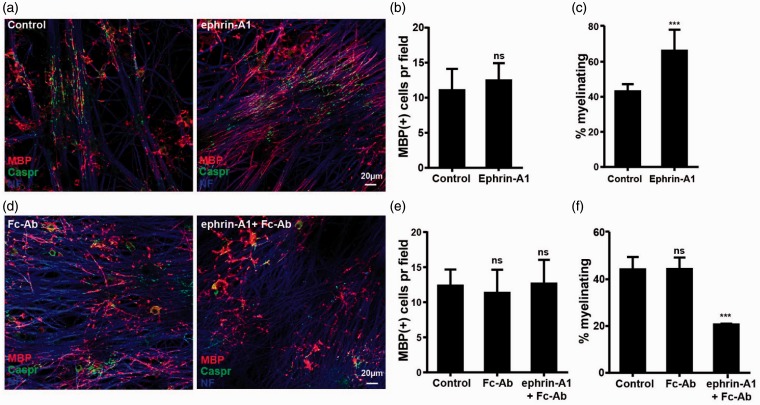
Forward EphA signaling in oligodendrocytes inhibits myelination. (a) The degree of myelination was analyzed after staining for Caspr (green), MBP (red), and NF (blue). Quantification of this experiment is shown in the following two panels, with means ± *SD* shown from at least 10 pictures from each of two coverslips in three independent experiments. (b) MBP + cells/20 × field. (c) Percentage of the MBP + cells forming at least two internode like structures and therefore scored as myelinating. (d–f) OPC and DRG neurons were cocultured in the absence or presence of ephrin-A1 preclustered with Fc-Ab as indicated. (d)The degree of myelination was analyzed after staining for Caspr (green), MBP (red), and NF (blue). Quantification of this experiment is shown in the next two panels, with means ± *SD* shown from at least 10 pictures from each of two coverslips in three independent experiments. (e) MBP + cells/20 × field. (f) Percentage of the MBP + cells forming at least two internode-like structures and therefore scored as myelinating. Statistical significance was tested by students *t* test (c and d) or one-way analysis of variance followed by a Dunnett’s multiple comparison tests (f and g). ***p < .001.

To analyze the role of EphA-ephrin-A signaling, we took advantage of the fact that soluble ephrin-A1 was not sufficient to reduce process extension in the oligodendrocytes (Figure 5(a) and (b)). This enabled us to use soluble ephrin-A1 as an antagonist to prevent bidirectional ephrin-A-EphA signaling. When soluble ephrin-A1 was added to the cocultures, we indeed observed a dramatic increase in the percentage of myelinating cells (Figure 8(a)–(c)). To test if this effect was required at early or late stages of the myelination process, oligodendrocytes were cocultured with DRG-neurons for 7 or 14 days prior to the addition of ephrin-A1 (Supplemental Figure 4). Interestingly, the stimulatory effect of ephrin-A1 was maintained when added at Day 7, but when added at Day 14, no effect was observed. This suggests that forward signaling through EphA receptors inhibit myelination, potentially by inducing process retraction and thereby preventing axo-glia contact formation at early stages of myelination. To further support that this was indeed the case, a similar experiment was carried out using ephrin-A1-Fc preclustered with Fc-antibodies, which is sufficient to induce forward signaling through EphA. As predicted, this inhibited normal myelination (Figure 8(d)–(f)) suggesting that downregulation of EphA forward signaling is required for axo-glia interaction and normal myelination. This is in line with our results obtained from oligodendrocytes cultured on an ephrin-A1 substrate clustered on the culture dish, which showed reduced ability of the oligodendrocytes to extend processes and form myelin sheets.

**Figure 9.**
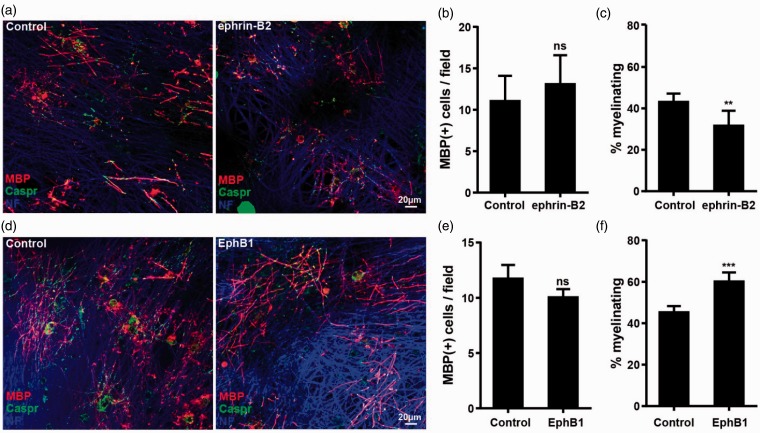
Forward and reverse Eph-ephrin-B signaling in oligodendrocytes have opposite effects on myelination. OPC were added to DRG neurons and cocultured in the absence or presence of soluble ephrin-B2, as indicated. (a) The degree of myelination was analyzed after staining for Caspr (green), MBP (red), and NF (blue). Quantification of this experiment is shown in the following two panels, with means ± *SD* shown from at least 10 pictures from each of two coverslips in three independent experiments. (b) MBP + cells/20 × field. (c) Percentage of the MBP + cells forming at least two internode-like structures and therefore scored as myelinating. (d, e) OPC and DRG neurons were cocultured in the absence or presence of soluble EphB1, as indicated. (d) The degree of myelination was analyzed after staining for Caspr (green), MBP (red), and NF (blue). Quantification of this experiment is shown in the next two panels, with means ± *SD* shown from at least 10 pictures from each of two coverslips in three independent experiments. (e) MBP + cells/20 × field. (f) Percentage of the MBP + cells forming at least two internode-like structures and therefore scored as myelinating. Statistical significance was tested by students *t* test. **p < .01; ***p < .001.

In contrast to ephrin-A1, the presence of soluble ephrin-B2 reduced the percentage of mature oligodendrocytes that myelinated (Figure 9(a)–(c)). This is in line with the ability of soluble ephrin-B2 to both inhibit integrin activation and block ephrin-B reverse signaling. Interestingly, ephrin-B2 was able to reduce myelination, even when added to the cultures at late time points, suggesting that EphB-ephrin-B signaling is also required late in the myelination process. In addition, soluble EphB1, which is able to block forward signaling and induce reverse signaling in the oligodendrocytes, enhanced myelination as would be predicted (Figure 9(d)–(f)). It is important to note that neither of the treatments significantly changed the number of cells in the cultures, which were positive for MAG, MBP, or NG2, further suggesting that the observed effects were caused by changes in axo-glia communication during the myelination process (Supplemental Figures 4, 5, and 6).

## Discussion

We have shown that a number of Ephs and ephrins are expressed in oligodendrocytes at different developmental stages, and that both forward and reverse signaling through these receptors have an impact on the ability of the oligodendrocytes to undergo morphological differentiation in culture and interact with neurons during the myelination process. Of particular interest, our results show distinct functions of forward signaling through the EphA and EphB receptors, and reverse signaling through ephrin-B. Forward signaling through EphA receptors reduced the ability of the oligodendrocytes to extend processes and form myelin sheets in culture. In addition, blocking of bidirectional signaling between ephrin-A1 and EphA receptors enhanced myelination in a coculture of oligodendrocytes and DRG neurons whereas stimulation of EphA receptors inhibited myelination. Combined, this suggest that EphA forward signaling into the oligodendrocyte causes process retraction, and thus prevents permanent axo-glia contact formation and initiation of myelination. We further showed that this effect of EphA is independent of the activational status of β1 integrins. By contrast, forward signaling through EphB only had an effect in the presence of the integrin ligand laminin, and the inhibitory effect of ephrin-B2 on myelin sheet formation could be reversed by overexpression of a constitutive active β1-integrin. This implies that in oligodendrocytes, ephrin-B2-induced forward signaling through EphB receptors is involved in controlling the activation of β1-integrins. In addition, we show that reverse signaling through ephrin-B, induced either by EphA4 or EphB1, stimulates myelin sheet formation, and finally the opposing effects of forward and reverse EphB-ephrin-B signaling were confirmed in the myelinating coculture system.

**Figure 10.**
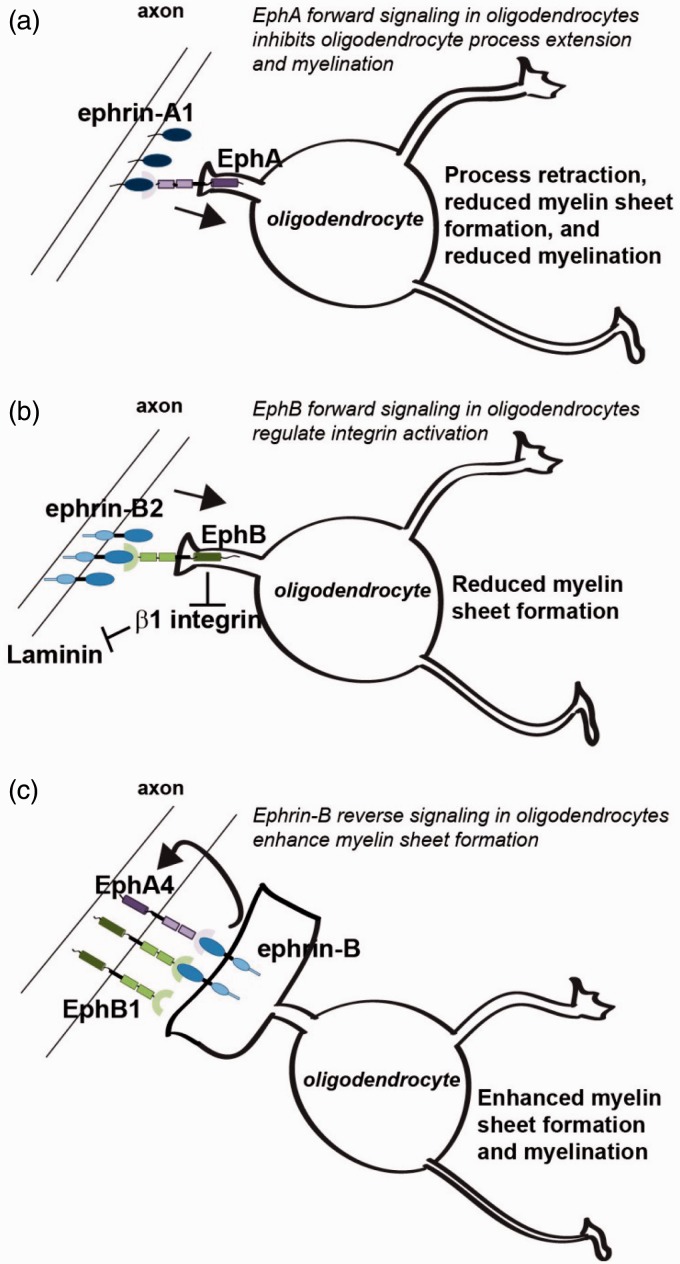
Working model: Opposing signals from Ephs and ephrins regulate axo-glia interaction and myelination. (a) EphA forward signaling in the oligodendrocyte inhibits oligodendrocyte process extension and myelination in an integrin independent manner. (b) EphB1 forward signaling in oligodendrocytes modulates integrin activation and thereby regulates axo-glia interactions and myelination. (c) Ephrin-B reverse signaling in oligodendrocytes, induced by EphB1 or EphA4, enhances myelin sheet formation. We propose that the specific combination of Ephs and ephrins, presented on the axonal surface, will determine whether a given axon becomes myelinated.

Together, this implies that the balance between forward and reverse Eph and ephrin signaling represents a possible mechanism of intercellular communication, controlling which axons are to be myelinated. We propose a working model in which negative regulatory guidance clues from ephrin-A and ephrin-B must be overcome by positive clues from EphA4 and EphB1, present on the axon surface, in order for the axons to be selected for myelination (Figure 10).

The existence of such an Eph-ephrin-guided selection process bares resemblance to the process of axonal guidance and growth cone dynamics (Xu and Henkemeyer, 2012), where the control of integrin-mediated adhesion also is required for decision making (Gupton and Gertler, 2010; Hines et al., 2010). We have previously shown that integrin activation is involved in controlling both the morphological differentiation of the oligodendrocytes and timely translation of MBP mRNA (Laursen et al., 2011). The latter suggests that a tight regulation of integrin activation is required to coordinate the wrapping process with MBP expression. This may explain why an increase in the level of the EphB1 receptor is necessary to control integrin activation in mature oligodendrocytes. In line with our findings, forward signaling through EphB has previously been reported to control integrin-mediated cell adhesion in other cell types through a mechanism involving phosphorylation of tyrosine-66 of R-Ras, causing decreased R-Ras activity (Zou et al., 1999). Interestingly, in oligodendrocytes, R-Ras has also been shown to regulate α6β1 integrin activation, which is required for process extension and myelin membrane formation in culture (Olsen and Ffrench-Constant, 2005). This implicates that the same signaling pathway is in use in the oligodendrocytes. The inhibitory effect of inactive R-Ras could be overcome by direct activation of the integrin with manganese ions (Olsen and Ffrench-Constant, 2005). Similarly, we show here that ephrin-B has no effect on cells expressing a constitutive active β1-integrin, and that the presence of laminin does not affect the ability of ephrin-B2 to induce EphB receptor phosphorylation, supporting the existence of a dynamically regulated system, where EphB signaling regulates the activation status of the *β*-1 integrin. Currently, the only factor known to induce integrin activation during the myelination process is laminin, present in the extracellular matrix surrounding the axons (Baron et al., 2005; Colognato et al., 2002; Olsen and Ffrench-Constant, 2005). This implicates that a balance between the amount of laminin in the extracellular matrix and ephrin-B2 on the axon surface controls the activation status of the integrin.

We also identified EphA4 and EphB1 as axonal adhesion molecules, which have a positive effect on the ability of the oligodendrocytes to extend myelin sheets. As a similar effect on myelin sheet formation was not observed with EphA2, this point to ephrin-B reverse signaling as a novel regulatory pathway during myelination. Interestingly, recent experiments have shown that EphA4 is present on hippocampal neurons at the time of myelination, and electron microscopy has further revealed clusters of EphA4 at contact points between axons and oligodendrocytes (Tremblay et al., 2009). This adds EphA4 and EphB1 to the list of very few axonal adhesion molecules, thought to be able to stimulate myelination. Previously, this list included only L1 (Laursen et al., 2009; Wake et al., 2011) and neuregulin (Brinkmann et al., 2008; Taveggia et al., 2008). A further understanding of how signals from these receptors integrate and overcome the negative regulatory signals will be essential for understanding the selection process. The observation that myelination is not completely inhibited in the absence of any one of these factors may suggest that the lack of one factor can be compensated for by upregulation of others.

We further identified ephrin-A1 as a novel negative regulatory molecule on the axonal surface, and we suggest that it may need to be downregulated for myelination to be initiated. Possible mechanisms behind such regulation could be cell surface shedding or reduced expression. Ephrin-A1-induced forward signaling was shown to prevent process extension from oligodendrocytes in an integrin-independent manner. In other systems, EphA forward signaling has been implicated in regulation of integrin activation (Bourgin et al., 2007; Miao et al., 2000) and has also been shown to directly regulate the activity of signaling molecules downstream of the integrins (Knoll and Drescher, 2004; Miao et al., 2000; Yamazaki et al., 2009). However, alternative downstream signaling pathways, controlling activation of the Rho family of GTPases, have also been implicated in EphA-mediated process retractions (Lisabeth et al., 2013). Even though we show that a constitutive active β1-integrin cannot reverse the inhibitory effect of ephrin-A1 on process extension, we cannot exclude that the EphA receptors may directly inhibit signaling molecules downstream of the β1-integrins. Interesting candidate molecules include ILK, FAK, and Src-family kinases, which are all known to be regulated by EphA receptors in other cell types (Knoll and Drescher, 2004; Miao et al., 2000; Yamazaki et al., 2009), and also known to be important for process extension in the oligodendrocytes (Camara et al., 2009; Chun et al., 2003; Colognato et al., 2004; Forrest et al., 2009; Liang et al., 2004; O’Meara et al., 2013).

Generally, the signaling pathways reported to be induced by forward and reverse signaling through the Eph-ephrin system are highly cell-type-specific. This is likely to reflect the specific expression profiles of receptor and ligands in adjacent cells at a given time point, and is further complicated by redundancy between family members and cis-interaction between ligands and receptors, reported to modulate the effect of trans-interactions (Carvalho et al., 2006; Kao and Kania, 2011). The observed upregulation of ephrin-A5 during oligodendrocyte differentiation may lead to inhibition of trans-signaling and thereby be an intrinsic mechanism by which the oligodendrocyte can overcome the inhibitory effect of EphA forward signaling.

In addition to the role of bidirectional Eph-ephrin signaling during axo-glia interactions and myelin sheath formation in the central nervous system we report here, others have suggested that the Eph/ephrin receptors may also play a role during migration of OPCs (Prestoz et al., 2004). It is interesting to note that Eph-ephrin signaling has also been reported to regulate Schwann cell migration (Afshari, et al., 2010). However, it is still an open question whether this signaling system also is involved in regulating axo-glia interactions in the peripheral nervous system.

Altogether our data suggest that Ephs and ephrins represent a novel family of adhesion molecules involved in controlling oligodendrocyte morphological differentiation and axo-glia interactions. This also points toward potentially novel ways, by which it will be possible to interfere with axo-glia interactions in order to enhance remyelination in demyelinating diseases, for example, multiple sclerosis. In particular, based on the enhanced myelination caused by soluble ephrin-A1 in cocultures, blocking of EphA forward signaling in the oligodendrocytes may be of specific interest. In support of the relevance and potential effect of such a strategy, ephrin-A1 and several EphA receptors were found to be upregulated in active lesions of multiple sclerosis patients (Sobel, 2005). Furthermore, recent data have shown that EphA4 knockout mice show a less severe clinical score after Myelin Oligodendrocyte Glycoprotein (MOG)-induced Experimental Autoimmune Encephalomyelitis (EAE) compared with wild-type animals (Munro et al., 2013).

## Summary

- Members of the Eph and ephrin subfamilies are upregulated during oligodendrocyte differentiation.
- Forward and reverse signaling through the Eph-ephrin receptors have opposite effects on process extension and myelin sheet formation in oligodendrocytes.
- Blocking of bidirectional EphA-ephrin-A signaling enhances myelination, whereas interfering with EphB-ephrin-B forward and reverse signaling in oligodendrocytes has opposite effects on myelination.
